# Supplementary material for: Microbiota mediated plasticity promotes thermal adaptation in the sea anemone Nematostella vectensis
Source: Nat Commun. 2022 Jul 1;13:3804. doi: 10.1038/s41467-022-31350-z (PMC9249911; doi:10.1038/s41467-022-31350-z)
Supplement: Supplementary file 1 — Supplementary Information [file 41467_2022_31350_MOESM1_ESM.pdf]

Supplementary Information

Microbiota mediated plasticity promotes thermal adaptation in the sea anemone *Nematostella vectensis*

Laura Baldassarre, Hua Ying, Adam M. Reitzel, Sören Franzenburg, Sebastian Fraune

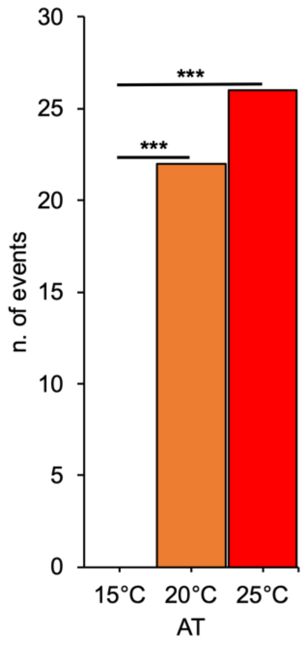

Figure S1. Number of spontaneous spawning events at each AT along the whole duration of the acclimation experiment (185 weeks). The spawning events were recorded per week when egg packs were found in all the boxes (n = 5) from each AT. Differences were tested through Fisher's Exact test (***p < 0.00001).

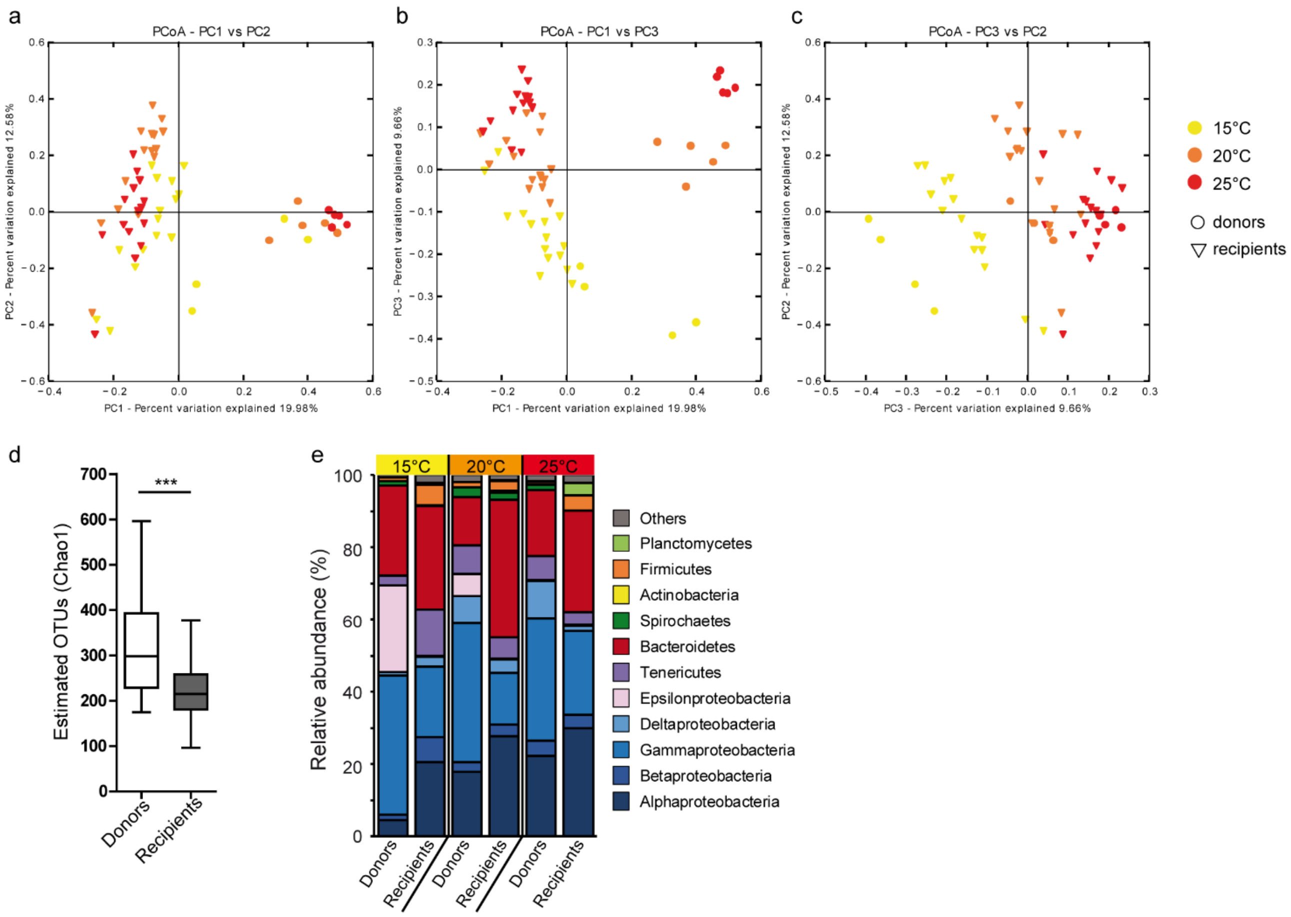

Figure S2. Bacterial transplantation from donor to recipient polyps. (a-c) PCoA (based on binary-Pearson metric, sampling depth = 3,600) illustrating similarity of donor and recipient bacterial communities based on AT of donor microbiota. (d) α-diversity (Chao1) comparison of donor and recipient bacterial communities (max rarefaction depth = 3,600), statistical analyses were performed using a t-test (two-tailed, df=57, ***$p < 0.001$), box plots are presenting center line, median; box limits, upper and lower quartiles; whiskers, 1.5x interquartile range. (e) Relative abundances of principal bacterial groups in donor and recipient samples, the abundances were summarized under the relative higher taxonomic categories (classes and phyla) and reported as percentages of the total.

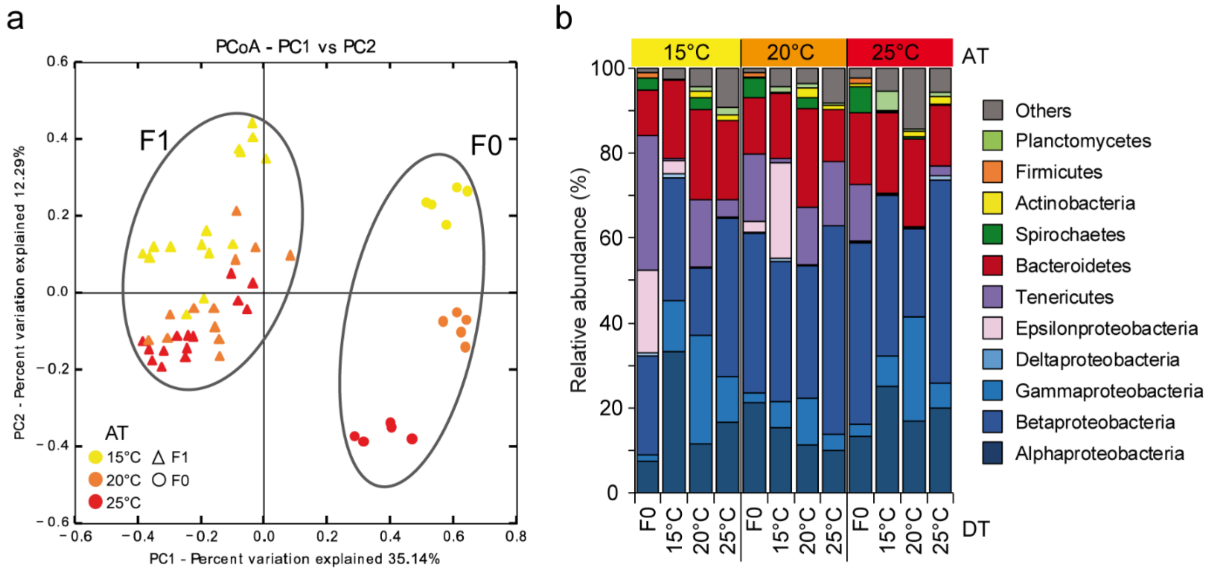

Figure S3. Transgenerational bacterial transmission from F0 to F1 animals. (a) PCoA (based on binary-Pearson metric, sampling depth = 24,500) illustrating similarity of bacterial communities of F0 and F1 animals (e) Relative abundances of principal bacterial groups in F0 and F1 samples, the abundances were summarized under the relative higher taxonomic categories (classes and phyla) and reported as percentages of the total.

Table S1. Statistical analysis if alphaproteobacterial abundances at 132 woa. A one-way ANOVA was performed followed by Bonferroni's post-hoc comparisons.

One-way analysis of variance				
P value	0,0004			
P value summary	***			
Are means signif. different? (P < 0.05)	Yes			
Number of groups	3			
F	17,27			
R squared	0,7585			
ANOVA Table	SS	df	MS	
Treatment (between columns)	0,07365	2	0,03682	
Residual (within columns)	0,02345	11	0,002132	
Total	0,0971	13		
Bonferroni's Multiple Comparison Test	Mean Diff.	t	P value	95% CI of diff
15°C vs 20°C	-0,1325	4,277	P < 0.01	-0.2198 to -0.04513
15°C vs 25°C	-0,1774	5,728	P < 0.001	-0.2648 to -0.09007
20°C vs 25°C	-0,04495	1,539	P > 0.05	-0.1273 to 0.03740

Table S2. Statistical analysis if epsilonproteobacterial abundances at 132 woa. A one-way ANOVA was performed followed by Bonferroni's post-hoc comparisons.

One-way analysis of variance				
P value	P<0.0001			
P value summary	***			
Are means signif. different? (P < 0.05)	Yes			
Number of groups	3			
F	25,96			
R squared	0,8252			
ANOVA Table	SS	df	MS	
Treatment (between columns)	0,1309	2	0,06543	
Residual (within columns)	0,02773	11	0,002521	
Total	0,1586	13		
Bonferroni's Multiple Comparison Test	Mean Diff.	t	P value	95% CI of diff
15°C vs 20°C	0,1769	5,254	P < 0.001	0.08197 to 0.2719
15°C vs 25°C	0,2364	7,018	P < 0.001	0.1414 to 0.3314
20°C vs 25°C	0,05944	1,872	P > 0.05	-0.03011 to 0.1490

Table S3. Statistical analysis determining the influence of AT on the bacterial colonization in recipient and donor polyps (number of permutations = 999).

		**Adonis**		**Anosim**	
**parameter**	**beta-diversity metric**	***R2***	***P value***	***R***	***P value***
**Donors' AT**	Binary-Pearson	0.102	0.001	0.211	0.001
	Bray-Curtis	0.083	0.001	0.123	0.001
	Pearson	0.075	0.001	0.082	0.001
	Weighted-Unifrac	0.060	0.002	0.078	0.002
	Unweighted-Unifrac	0.091	0.001	0.193	0.001

Table S4. Bacterial OTUs overrepresented at 15 and 25°C in both acclimated and recolonized animals. Through LEfSe analyses OTUs were detected that are significantly overrepresented at 15 vs 25°C for both the acclimated and the transplanted ones. For the statics a factorial Kruskal-Wallis test (α-value = 0.05) followed by pairwise Wilcoxon rank-sum tests (α-value = 0.05) were applied. To estimate the effect size, a LDA analysis (logarithmic score threshold = 2) was performed. In the table The OTUs are reported that are shared between the acclimated and the transplanted animals according with the AT (in grey the classifications that don't reach 80% confidence similarity with reference sequences available in public databases). (p-values and LDA scores are reported in Suppl. Data 3).

Phylum	Class	Order	Family	Genus	OTU	AT
Proteobacteria [100%]	Betaproteobacteria [100%]	Burkholderiales [100%]	Alcaligenaceae [100%]	*Castellaniella* [100%]	509480	15°C
Proteobacteria [100%]	Gammaproteobacteria [100%]	Alteromonadales [96%]	Colwelliaceae [96%]	*Colwellia* [86%]	349769	
Proteobacteria [100%]	Gammaproteobacteria [100%]	Cellvibrionales [100%]	Spongiibacteraceae [98%]	*Marortus* [87%]	328	
Planctomycetes [100%]	Phycisphaerae [100%]	Phycisphaerales [100%]	Phycisphaeraceae [100%]	*Algisphaera* [74%]	135	25°C
Bacteroidetes [100%]	Flavobacteriia [100%]	Flavobacteriales [100%]	Flavobacteriaceae [100%]	*Muricauda* [98%]	129	
Proteobacteria [99%]	Alphaproteobacteria [97%]	Emcibacterales [69%]	Emcibacteraceae [69%]	*Emcibacter* [69%]	42	
Proteobacteria [100%]	Alphaproteobacteria [100%]	Rhodobacterales [100%]	Rhodobacteraceae [100%]	*Sulfitobacter* [98%]	650063	
Proteobacteria [100%]	Alphaproteobacteria [100%]	Rhodobacterales [100%]	Rhodobacteraceae [100%]	*Sulfitobacter* [58%]	162	
Proteobacteria [100%]	Betaproteobacteria [100%]	Nitrosomonadales [89%]	Methylophilaceae [83%]	*Methylotenera* [60%]	232	
Proteobacteria [100%]	Gammaproteobacteria [100%]	Thiotrichales [100%]	Francisellaceae [100%]	*Francisella* [100%]	144057	
Proteobacteria [100%]	Gammaproteobacteria [100%]	Thiotrichales [100%]	Francisellaceae [100%]	*Francisella* [100%]	218	
Proteobacteria [100%]	Gammaproteobacteria [100%]	Oceanospirillales [100%]	Oceanospirillaceae [100%]	*Neptunomonas* [78%]	275	
Proteobacteria [100%]	Gammaproteobacteria [100%]	Vibrionales [100%]	Vibrionaceae [100%]	*Vibrio* [100%]	939811	

Table S5. Statistical analysis determining the influence of AT on bacterial colonization in F0 and F1 polyps (number of permutations = 999).

		**Adonis**		**Anosim**	
**parameter**	**beta-diversity metric**	***R2***	***P value***	***R***	***P value***
**F0 AT**	Binary-Pearson	0.131	0.001	0.200	0.001
	Bray-Curtis	0.098	0.001	0.152	0.001
	Pearson	0.098	0.001	0.117	0.001
	Weighted-Unifrac	0.063	0.053	0.051	0.031
	Unweighted-Unifrac	0.119	0.001	0.163	0.001

Table S6. Bacterial OTUs overrepresented in both F0 and F1 animals. Through LEfSe analyses OTUs were detected that are significantly overrepresented both in F0 and F1 animals For the statics a factorial Kruskal-Wallis test (α-value = 0.05) followed by pairwise Wilcoxon rank-sum tests (α-value = 0.05) were applied. To estimate the effect size, a LDA analysis (logarithmic score threshold = 2) was performed. In the table, the OTUs are reported that are shared between the F0 and the F1 animals according with the AT in the different developmental temperatures (in grey the classifications that do not reach 80% confidence similarity with reference sequences available in public databases). (p-values and LDA scores are reported in Suppl. Data 4).

AT	Phylum	Class	Order	Family	Genus	OTU	DT
15°C	Bacteroidetes [100%]	Flavobacteriia [100%]	Flavobacteriales [100%]	Cryomorphaceae [91%]	*Salinirepens* [55%]	153	15°C
	Bacteroidetes [100%]	Flavobacteriia [100%]	Flavobacteriales [100%]	Flavobacteriaceae [100%]	*Polaribacter* [53%]	168	
	Bacteroidetes [98%]	Saprospiria [95%]	Saprospirales [95%]	Lewinellaceae [92%]	*Lewinella* [84%]	84	
	Proteobacteria [100%]	Alphaproteobacteria [100%]	Rhodobacterales [100%]	Rhodobacteraceae [100%]	*Celeribacter* [100%]	159906	
	Proteobacteria [100%]	Alphaproteobacteria [100%]	Rhodobacterales [100%]	Rhodobacteraceae [100%]	*Celeribacter* [60%]	20352	
	Proteobacteria [100%]	Alphaproteobacteria [100%]	Rhodobacterales [100%]	Rhodobacteraceae [100%]	*Pseudorhodobacter* [100%]	168456	
	Campilobacterota [100%]	Campylobacteria [100%]	Campylobacterales [100%]	Arcobacteraceae [100%]	*Pseudarcobacter* [95%]	178	
	Bacteroidetes [100%]	Flavobacteriia [100%]	Flavobacteriales [100%]	Cryomorphaceae [91%]	*Salinirepens* [55%]	153	20°C
	Bacteroidetes [98%]	Saprospiria [95%]	Saprospirales [95%]	Lewinellaceae [92%]	*Lewinella* [84%]	84	
	Proteobacteria [100%]	Alphaproteobacteria [100%]	Rhodobacterales [100%]	Rhodobacteraceae [100%]	*Pseudorhodobacter* [100%]	168456	
	/	/	/	/	/	/	25°C
20°C	Proteobacteria [100%]	Gammaproteobacteria [100%]	Oceanospirillales [97%]	Oceanospirillaceae [96%]	*Bacterioplanoides* [35%]	75	15°C
	Proteobacteria [100%]	Gammaproteobacteria [100%]	Pseudomonadales [100%]	Pseudomonadaceae [100%]	*Pseudomonas* [100%]	543864	
	Proteobacteria [100%]	Gammaproteobacteria [100%]	Pseudomonadales [100%]	Pseudomonadaceae [100%]	*Pseudomonas* [88%]	750018	
	Bacteroidetes [100%]	Flavobacteriia [100%]	Flavobacteriales [100%]	Flavobacteriaceae [95%]	*Paramesonia* [37%]	94	20°C
	Bacteroidetes [100%]	Saprospiria [100%]	Saprospirales [100%]	Saprospiraceae [100%]	*Aureispira* [100%]	213	
	/	/	/	/	/	/	25°C
25°C	Proteobacteria [100%]	Gammaproteobacteria [100%]	Oceanospirillales [96%]	Oceanospirillaceae [96%]	*Oleibacter* [96%]	543999	15°C
	Proteobacteria [100%]	Gammaproteobacteria [100%]	Alteromonadales [100%]	Alteromonadaceae [100%]	*Alteromonas* [100%]	1106960	
	Proteobacteria [96%]	Alphaproteobacteria [95%]	Emcibacterales [29%]	Emcibacteraceae [29%]	*Emcibacter* [29%]	136	20°C
	Proteobacteria [100%]	Gammaproteobacteria [100%]	Pseudomonadales [89%]	Pseudomonadaceae [88%]	*Pseudomonas* [85%]	11301	
	Proteobacteria [100%]	Gammaproteobacteria [100%]	Nevskiales [100%]	Nevskiaceae [100%]	*Polycyclovorans* [100%]	2162897	
	Proteobacteria [100%]	Gammaproteobacteria [100%]	Oceanospirillales [96%]	Oceanospirillaceae [96%]	*Oleibacter* [96%]	543999	25°C

Table S7. RNA-seq samples and read numbers

Sequencing run	Read length	IKMD ID	SAMPLE name	Raw read pairs	Trimming: Read pairs retained	Trimming: retained %	Mapping: Reads mapped	Mapping: mapped %
RUN_001	75	H26641	15_1	21965881	20156341	91.76	9486045	47.06
		H26642	20_1	26272441	23888905	90.93	16538026	69.23
		H26643	25_1	27445720	24825197	90.45	18833605	75.86
		H26644	15_2	22016275	20159528	91.57	10033196	49.77
		H26645	20_2	21193794	19438871	91.72	10417257	53.59
		H26646	25_2	28770569	26617808	92.52	12373203	46.48
		H26647	15_3	20617657	19044009	92.37	6639169	34.86
		H26648	20_3	20717968	19134074	92.35	10478621	54.76
		H26649	25_3	19915554	18291356	91.84	9743552	53.27
		H26650	15_4	27972644	25655145	91.72	12019124	46.85
		H26651	20_4	17849537	16260590	91.1	11263333	69.27
		H26652	25_4	19056424	17458322	91.61	10229475	58.59
		H26653	15_5	22700911	20856028	91.87	10274581	49.26
		H26654	20_5	28615590	26324793	91.99	17221169	65.42
		H26655	25_5	19402205	17622792	90.83	11791982	66.91
RUN_002	150	H26641	15_1	18423491	14498669	78.7	8981800	61.95
		H26642	20_1	20282809	16073444	79.25	12144504	75.56
		H26643	25_1	20518674	16343745	79.65	13310392	81.44
		H26644	15_2	22047499	17536766	79.54	11378824	64.89
		H26645	20_2	19248002	14005749	72.76	9156279	65.38
		H26646	25_2	15945068	12264170	76.92	7457059	60.80
		H26647	15_3	20934329	16338633	78.05	8873633	54.31
		H26648	20_3	20379189	15838885	77.72	10386438	65.58
		H26649	25_3	20571296	16103120	78.28	10650106	66.14
		H26650	15_4	19930969	15926267	79.91	9873708	62.00
		H26651	20_4	21426288	16883302	78.8	12716328	75.32
		H26652	25_4	21070974	16272134	77.23	10720893	65.88
		H26653	15_5	16800860	12525327	74.55	7769274	62.03
		H26654	20_5	20133696	15621338	77.59	11299661	72.33
		H26655	25_5	20342454	15823349	77.78	11885520	75.11

Table S8. Statistics of Ensembl and updated gene annotation

	Annotation	Ensembl (v1.0)	updated
Genome and Genes	assembly Size (Mb)	356	356
	No.genes	24.773	27.527
	genicSize (Mb)	114	144
	% genic	32,02	40,45
Gene length	mean	4.609	5.459
	median	2.473	2.716
	longest	97.791	194.300
mRNA length	mean	1.166	1.474
	median	837	921
	longest	33.620	33.620
Exon length	mean	219	254
	median	123	127
	longest	33.055	33.055
Intron length	mean	797	829
	median	437	436
	longest	48.615	101.549
	Total size (kb)	85.252	106.280

Table S9. Numbers of BUSCO v5 metazoa_odb10 genes (n = 954) identified

	Ensembl	updated
Complete (Duplicated)	858 (17)	890 (32)
Fragmented	47	36
Missing	49	28

Table S10. Numbers of DEGs identified from DESeq2 and Voom.

compare	direction	DESeq2	voom	common	% DESeq2	% voom
High_Low	UP	3.594	4.351	3.589	99,86	82,49
High_Low	DOWN	3.870	4.214	3.778	97,62	89,65
High_Med	UP	1.380	1.764	1.355	98,19	76,81
High_Med	DOWN	1.831	2.112	1.769	96,61	83,76
Med_Low	UP	2.633	3.255	2.626	99,73	80,68
Med_Low	DOWN	2.873	3.201	2.784	96,90	86,97
