# Supplementary material for: Microbiota mediated plasticity promotes thermal adaptation in the sea anemone Nematostella vectensis
Source: Nat Commun. 2022 Jul 1;13:3804. doi: 10.1038/s41467-022-31350-z (PMC9249911; doi:10.1038/s41467-022-31350-z)
Supplement: Supplementary file 3 — Description of Additional Supplementary Files [file 41467_2022_31350_MOESM3_ESM.docx]

**Description of Additional Supplementary Files**

**Supplementary Data 1.** Enriched GO terms that were identified by DEGs from both DeSeq2 and Voom comparing 15 °C and 25°C samples.

**Supplementary Data 2.** Presence/absence comparison of OTUs in donor and recipient samples based on sequencing reads. (+) present in both samples, (D) present only in donors, (R) present only in recipients, (-) not present in both samples.

**Supplementary Data 3.** Presence/absence comparison of OTUs in F0 and F1 samples based on sequencing reads. (+) present in both samples, (F0) present only in parents, (F1) present only in offspring, (-) not present in both samples.

**Supplementary Data 4.** Updated gene models used in this study.
