# Supplementary material for: Microbiota mediated plasticity promotes thermal adaptation in the sea anemone Nematostella vectensis
Source: Nat Commun. 2022 Jul 1;13:3804. doi: 10.1038/s41467-022-31350-z (PMC9249911; doi:10.1038/s41467-022-31350-z)
Supplement: Supplementary file 8 — Reporting Summary [file 41467_2022_31350_MOESM8_ESM.pdf]

nature portfolio

Corresponding author(s): Sebastian Fraune

Last updated by author(s): Apr 28, 2022

Reporting Summary

Nature Portfolio wishes to improve the reproducibility of the work that we publish. This form provides structure for consistency and transparency in reporting. For further information on Nature Portfolio policies, see our Editorial Policies and the Editorial Policy Checklist.

Statistics

For all statistical analyses, confirm that the following items are present in the figure legend, table legend, main text, or Methods section.

n/a	Confirmed	
☐	☒	The exact sample size (*n*) for each experimental group/condition, given as a discrete number and unit of measurement
☐	☒	A statement on whether measurements were taken from distinct samples or whether the same sample was measured repeatedly
☐	☒	The statistical test(s) used AND whether they are one- or two-sided<br>*Only common tests should be described solely by name; describe more complex techniques in the Methods section.*
☒	☐	A description of all covariates tested
☐	☒	A description of any assumptions or corrections, such as tests of normality and adjustment for multiple comparisons
☐	☒	A full description of the statistical parameters including central tendency (e.g. means) or other basic estimates (e.g. regression coefficient) AND variation (e.g. standard deviation) or associated estimates of uncertainty (e.g. confidence intervals)
☐	☒	For null hypothesis testing, the test statistic (e.g. *F*, *t*, *r*) with confidence intervals, effect sizes, degrees of freedom and *P* value noted<br>*Give P values as exact values whenever suitable.*
☒	☐	For Bayesian analysis, information on the choice of priors and Markov chain Monte Carlo settings
☒	☐	For hierarchical and complex designs, identification of the appropriate level for tests and full reporting of outcomes
☒	☐	Estimates of effect sizes (e.g. Cohen's *d*, Pearson's *r*), indicating how they were calculated

Our web collection on statistics for biologists contains articles on many of the points above.

Software and code

Policy information about availability of computer code

Data collection	no software for data collection was used
Data analysis	QIIME 1.9.0; JASP v0.16; LEfSe; FastQC v0.11.7; Trimmomatic v.0.38; hisat2 v2.1.0; StringTie v2.0;<br>Scallop v0.10.4; TACO; gffCompare v0.11.2;BUSCO v5.2.2; Subread-2.0.0; DESeq2 v1.28.1;<br>BioConductor package; limma voom v3.44.3; package<br>topGO v2.40.0

For manuscripts utilizing custom algorithms or software that are central to the research but not yet described in published literature, software must be made available to editors and reviewers. We strongly encourage code deposition in a community repository (e.g. GitHub). See the Nature Portfolio guidelines for submitting code & software for further information.

Data

Policy information about availability of data

All manuscripts must include a data availability statement. This statement should provide the following information, where applicable:
- Accession codes, unique identifiers, or web links for publicly available datasets
- A description of any restrictions on data availability
- For clinical datasets or third party data, please ensure that the statement adheres to our policy

Transcriptomic data and 16S rRNA gene sequencing data are available at the NCBI database under accession codes GSE168938 [https://www.ncbi.nlm.nih.gov/geo/query/acc.cgi?acc=GSE168938] and PRJNA742683 [https://www.ncbi.nlm.nih.gov/sra/?term=PRJNA742683].

Field-specific reporting

Please select the one below that is the best fit for your research. If you are not sure, read the appropriate sections before making your selection.

☒ Life sciences ☐ Behavioural & social sciences ☐ Ecological, evolutionary & environmental sciences

For a reference copy of the document with all sections, see nature.com/documents/nr-reporting-summary-flat.pdf

Life sciences study design

All studies must disclose on these points even when the disclosure is negative.

Sample size	No sample size calculation was performed. In the long-term acclimation experiments, 3 x 5 replicate animal cultures (50 clonal animals each, 750 animals in total) were kept for more than 3 years. This decision was based on our long-term experience working with clonal animals without genetic variability and proved to be sufficient to detect biological variation.
Data exclusions	no data were excluded
Replication	all experiments are based on a long-term experiment with independent replicates (n=5). The long-term experiment itself was not repeated. All performed experiments are given in the manuscript. All the results reported are the outcomes of all the replications performed and all attempts at replication were successful.
Randomization	experimental groups were randomized
Blinding	In the experiments involving heat stress, the animals were blinded. Other experiments involving the analysis of DNA and RNA sequences depend on the metadata of the samples and were therefore not blinded.

Reporting for specific materials, systems and methods

We require information from authors about some types of materials, experimental systems and methods used in many studies. Here, indicate whether each material, system or method listed is relevant to your study. If you are not sure if a list item applies to your research, read the appropriate section before selecting a response.

Materials & experimental systems

n/a	Involved in the study	
☒	☐	Antibodies
☒	☐	Eukaryotic cell lines
☒	☐	Palaeontology and archaeology
☐	☒	Animals and other organisms
☒	☐	Human research participants
☒	☐	Clinical data
☒	☐	Dual use research of concern

Methods

n/a	Involved in the study	
☒	☐	ChIP-seq
☒	☐	Flow cytometry
☒	☐	MRI-based neuroimaging

Animals and other organisms

Policy information about studies involving animals; ARRIVE guidelines recommended for reporting animal research

Laboratory animals	Nematostella vectensis (Stephenson 1935). The adult animals of the laboratory culture were F1 offspring of CH2XCH6 individuals collected from the Rhode River in Maryland. The acclimated and recolonized animals were all female clones. Two males have been used for fertilization of oocytes. The sexes of the offspring were not determinable. The age of the adults was > 13 years at the beginning of the experiment; the juveniles have been sacrificed after one month.
Wild animals	The study did not involved wild animals
Field-collected samples	Males and females of the initial culture of N. vectensis were a kind gift of Ulrich Technau (Department of Molecular Evolution and Development, University Vienna), which were F1 offspring of previously collected specimens from the Rhode River in Maryland (Hand and Uhlinger 1992). They were kept under constant, artificial conditions without substrate or light in plastic boxes filled with ca. 1L. Nematostella Medium (NM), which was adjusted to 16‰ salinity with Red Sea Salt® and Millipore H2O. Polyps were fed 2 times a week with first instar nauplius larvae of Artemia salina as prey (Ocean Nutrition Micro Artemia Cysts 430 - 500 gr, Coralsands, Wiesbaden, Germany) and washed once a week.
Ethics oversight	no ethical approval was required as we worked with cnidarian animals

Note that full information on the approval of the study protocol must also be provided in the manuscript.
